# Reversible predictors of reversion from mild cognitive impairment to normal cognition: a 4-year longitudinal study

**DOI:** 10.1186/s13195-019-0480-5

**Published:** 2019-03-13

**Authors:** Hiroyuki Shimada, Takehiko Doi, Sangyoon Lee, Hyuma Makizako

**Affiliations:** 1 Department of Preventive Gerontology, Center for Gerontology and Social Science, National Center for Geriatrics and Gerontology, 7-430 Morioka-cho, Obu City, Aichi Prefecture 474-8511 Japan; 2 Department of Physical Therapy, School of Health Sciences, Faculty of Medicine, Kagoshima University, Kagoshima City, Japan

**Keywords:** Mild cognitive impairment, Lifestyle, Activities, Risk, Dementia

## Abstract

**Background:**

Although previous studies have revealed many factors related to mild cognitive impairment (MCI) reversion, information about reversible factors related MCI reversion is limited, impeding the development of intervention strategies. The aim of the present study was to examine whether reversible factors such as lifestyle activities are associated with MCI reversion in elderly individuals using the National Center for Geriatrics and Gerontology—Study of Geriatric Syndromes database. A total of 396 community-living older adults (age ≥ 65 years) participated in the study. They were classified as reverters or non-reverters from mild cognitive impairment to normal cognition. We assessed lifestyle activities, potential confounding factors of cognitive decline, and reversion of mild cognitive impairment.

**Results:**

In a completed data set of 396 participants, 202 participants (51.0%) reverted from MCI to normal cognition. The reversion rate in participants for whom we imputed data was 34.3%. In the imputed group, a logistic regression model showed that the odds ratios (ORs) for reversion were significantly higher in participants who drove a car (OR 1.50, 95% confidence interval (CI) 1.41–1.60), used a map to travel to unfamiliar places (OR 1.12, 95% CI 1.06–1.18), read books or newspapers (OR 1.54, 95% CI 1.37–1.73), took cultural classes (OR 1.10, 95% CI 1.04–1.15), attended meetings in the community (OR 1.22, 95% CI 1.16–1.28), participated in hobbies or sports activities (OR 1.09, 95% CI 1.03–1.16), and engaged in fieldwork or gardening (OR 1.14, 95% CI 1.08–1.21). The imputed sample showed that non-reverters were more likely to discontinue fieldwork or gardening (11.0% vs. 6.1%) than reverters during the follow-up period.

**Conclusions:**

Specific lifestyle activities may play important roles in MCI reversion in older adults. The longitudinal data indicate that it is reasonable to recommend that individuals continue to engage in fieldwork or gardening to increase their chance of recovery from MCI.

**Electronic supplementary material:**

The online version of this article (10.1186/s13195-019-0480-5) contains supplementary material, which is available to authorized users.

## Background

Mild cognitive impairment (MCI) is considered to be an intermediate state between normal cognitive aging and early dementia [1]. Identifying individuals with MCI can facilitate the timely detection of dementia and may uncover useful information regarding targets for prevention in the community [2]. However, the significance of an MCI categorization is uncertain given the longitudinal data suggesting that MCI may be unstable, as many individuals are found to be cognitively normal in follow-up assessments. A meta-analysis that assessed reversion rates in 25 studies indicated an overall reversion rate of approximately 24% [3]. Subject-based factors include recovery from illness, differing measurement, and diagnosis methodologies as well as variations in the cutoff scores used to diagnose MCI may also increase the probability of reversion from MCI to normal cognition [4–6].

Although a number of previous studies have suggested that MCI is unstable, this is especially evident from studies of MCI patients presenting to memory disorder clinics, in whom the annual rate of progression to dementia is reported to range from 10 to 15% [7]. While rates of progression have been found to be lower in population-based studies, i.e., between 6 and 10%, these values are higher than the 1 to 2% annualized incidence rates of dementia in the general older population [8]. Thus, we believe that recovery from MCI to normal cognition has important implications for the prevention of dementia.

The reduction of risk factors and promotion of protective factors is essential to the formulation of effective interventions for preventing dementia. Prospective observational studies have suggested a number of common factors related to MCI reversion. These include younger age, being unmarried, APOE ε4 status, neuroimaging factors (i.e., larger hippocampal volumes), better cognitive function, MCI subtype, absence of an informant-based memory complaint, arthritis, high blood pressure and lipid abnormality, high mental activity, openness to experience, better vision, and better smelling ability [9–13]. Although previous studies have revealed many factors related to MCI reversion, information about reversible factors related MCI reversion is limited, impeding the development of intervention strategies.

The aim of the present study was to examine whether reversible factors such as lifestyle activities are associated with MCI reversion in elderly individuals using the National Center for Geriatrics and Gerontology—Study of Geriatric Syndromes (NCGG-SGS) database [14]. Previous studies have suggested that elderly persons who had participated to a greater extent in everyday activities had a lower risk of dementia compared with those who had participated to a lesser extent [15–23]. Although these studies identified the efficacy of leisure-time activities including physical, cognitive, and social activities, the relationships between dementia incidence and lifestyle activities, such as visiting friends and house cleaning, are not well known. We hypothesized that participants who completed certain everyday activities would have a higher proportion of MCI reversion compared with those who had not participated in such activities.

## Methods

### Participants

Participants were selected from adults enrolled in a population-based cohort study titled “The Obu Study of Health Promotion for the Elderly (OSHPE)” [24], which is part of the NCGG-SGS [14]. In the present study, we analyzed longitudinal data from 396 community-dwelling older adults who were ≥ 65 years old (mean age 71.1 ± 4.5 years, 181 men and 215 women), had participated in both the first and second waves of the OSHPE, and did not have MCI at the time of the first wave assessment. The first wave of the OSHPE was held between August 2011 and February 2012. During this wave, 5104 community-dwelling elderly people participated in a baseline OSHPE assessment. Of these, 3095 (60.6%) took part in a second-wave cognitive examination between August 2015 and August 2016.

**Fig. 1:**
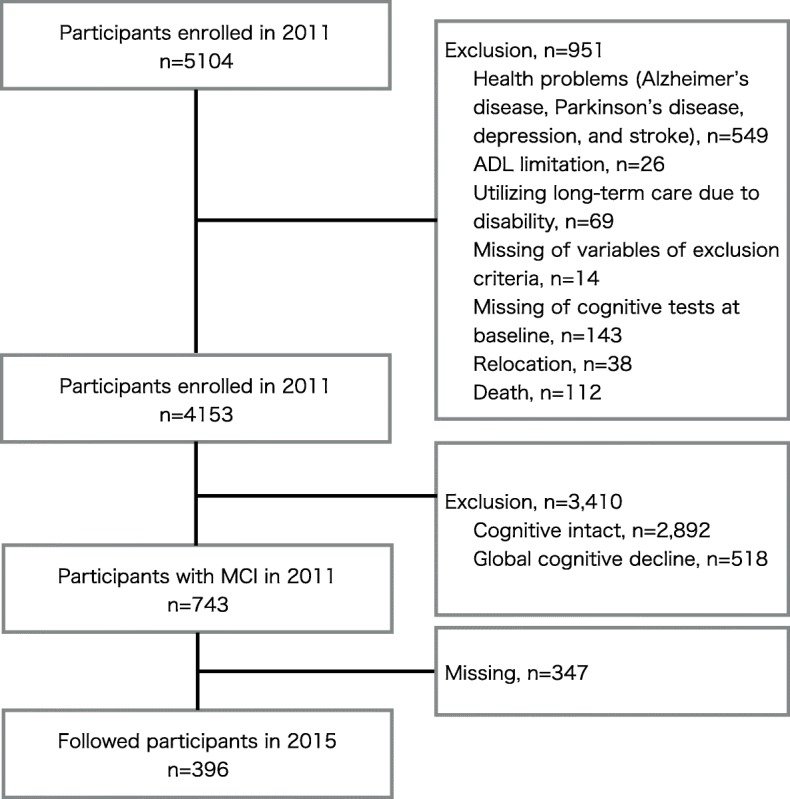
Flow diagram of sample selection

The inclusion criteria were residence in Obu and aged ≥ 65 years at the time of the first examination (August 2011 to March 2013). The baseline exclusion criteria were health problems such as Alzheimer’s disease, Parkinson’s disease, depression, or stroke (*n* = 549); inability to perform basic tasks of daily living such as eating, grooming, bathing, and climbing up and down stairs (*n* = 26); need for support or care as certified by the Japanese public long-term care insurance system due to disability (*n* = 69); missing data regarding the exclusion criteria (*n* = 14); inability to complete cognitive tests at the baseline assessment (*n* = 143); relocation (*n* = 38) or death (*n* = 112) during the follow-up period; normal cognition (*n* = 2892); and global cognitive decline at the baseline assessment (*n* = 518). Of the 743 potential participants, 347 did not receive the second-wave cognitive examination (Fig. 1). Thus, we analyzed data from 396 participants. Multiple imputation was used to adjust for selection bias and loss of information, because we found the potential bias of the sample in baseline data on those who remained versus left the follow-up (Additional file 1). We imputed reversion status, which was divided into reverters, who recovered from MCI to normal cognition, and non-reverters, who had MCI, global cognitive decline (as indicated by a Mini-Mental State Examination (MMSE) [25] score of < 24) and/or Alzheimer’s disease (AD), for participants with missing data. We generated 50 imputed values for each participant with missing data using a fully conditional specification multiple imputation procedure assuming that those values were missing at random [26]. The fully conditional specification procedure first substituted missing data of potential predictors by plausible values using an iterative stochastic algorithm, which results in 50 multiple complemented replications of the original dataset. The main advantages of using multiple imputation over the complete sample were as follows: (1) increased power to detect associations in a multiple regression model using the partial information available for some participants and (2) this enabled us to account for the likely possibility that the presence of missing scores was not completely random, but that, among participants with similar known characteristics, the distribution of missing values would resemble that of known values [10]. All participants gave their informed consent before they were included in the study. The study protocol was approved by an institutional review board.

### Measurements of lifestyle activity

Participants completed a questionnaire that comprised 16 questions regarding instrumental activities of daily living (IADL), cognitive activity, social activity, and productive activity as different elements of lifestyle activity. The questionnaire included the following questions that measured IADL: (1) “Do you go outdoors using the bus and train?,” (2) “Do you engage in cash handling and banking?,” (3) “Do you drive a car?,” and (4) “Do you use maps to go to unfamiliar places?.” Items that measured cognitive activity included (5) “Do you read books or newspapers?,” (6) “Do you engage in cognitive stimulation such as board games and learning?,” (7) “Do you engage in cultural classes?,” and (8) “Do you use a personal computer?.” Items that measured social activity included (9) “Do you talk with other people every day?,” (10) “Are you sometimes called on for advice?,” (11) “Do you attend meetings in the community?,” and (12) “Do you engage in hobbies or sports activities?.” Items that measured productive activity included (13) “Do you engage in housecleaning?,” (14) “Do you engage in fieldwork or gardening?,” (15) “Do you take care of a grandchild or pet?”, and (16) “Do you engage in paid work?.” Answers of “yes” were determined to be positive responses.

### Measurement of cognitive functions and incident AD

We used the National Center for Geriatrics and Gerontology-Functional Assessment Tool (NCGG-FAT), which is an iPad application, to conduct cognitive screening [27]. The NCGG-FAT includes the following domains: (1) memory (wordlist memory-I [immediate recognition] and word list memory-II [delayed recall]); (2) attention (a tablet version of the Trail Making Test-part A; TMT-part A); (3) executive function (a tablet version of the TMT-part B), and (4) processing speed (a tablet version of the Digit Symbol Substitution Test). The NCGG-FAT has been shown to have high test–retest reliability and moderate to high criterion-related validity [28] and predictive validity [29] among community-dwelling older persons. Well-trained study assistants conducted the assessments of cognitive functioning. Before the study began, all staff received training from the authors regarding the protocols for administering the assessments. Potential participants with MCI were identified after reviewing available clinical, neuropsychological, and laboratory data at meetings involving study neurologists and neuropsychologists, as previously described [24]. In brief, the MCI participants were independently recruited using the NCGG-FAT, which has two memory tasks, tests of attention and executive function, and a processing speed task. Using established criteria [1], we diagnosed MCI in individuals who exhibited cognitive impairment but were functionally independent in terms of basic daily life activities. For all cognitive tests, we used established standardized thresholds in each corresponding domain for defining impairment in population-based cohorts comprising community-dwelling older persons (scores > 1.5 standard deviations [SDs] below the age- and education-specific means). We used the MMSE to measure global cognitive function [25]. Specifically, we used < 24 points on the MMSE as a cutoff score for global cognitive impairment (GCI), in accordance with previous findings [30]. Participants whose scores were all > 1.5 SD units above the mean were classified as belonging to the normal cognition (NC) group.

In the present study, participants were tracked monthly for newly incident AD, as recorded by the Japanese National Health Insurance and Later-Stage Medical Care systems [31]. Participants were considered to have AD if they had been diagnosed by a medical doctor according to the International Classification of Diseases, 10th revision. Incidence of AD was based simply on diagnoses by third-party doctors who were blinded to the design and participant groups of the study. Participants were divided into the following two groups: the reverter group, who had CN at the follow-up, and the non-reverter group, who had MCI, GCI, and/or AD at the follow-up.

### Potential confounding factors

Demographic variables, chronic medical conditions, psychosocial factors, and physical performance are associated with cognitive decline in older persons [32, 33]. All multivariate models included the following covariates unless otherwise specified: age at enrollment, sex, educational level, current smoking status, chronic medical illnesses, depressive symptoms, and comfortable walking speed. The presence of the following self-reported chronic medical illnesses was entered into the models: heart disease, pulmonary disease, hypertension, and diabetes mellitus. Depressive symptoms were measured using the 15-item Geriatric Depression Scale (GDS) [34]. We calculated average walking speed by asking the participants to complete five trials in which they walked at a comfortable walking speed.

### Statistical analysis

We used Student’s *t* tests and Pearson’s chi-squared test to examine differences in the baseline participant characteristics and between the reverter and non-reverter groups. We used the chi-squared test with adjusted standardized residuals to determine whether MCI status significantly affected reversion. Residuals followed the *t* distribution: *t* > 1.96 was accepted as indicating *P* < .05, and *t* > 2.56 as indicating *P* < .01.

We calculated the reversion rates from MCI to NC and non-reversion rates during the follow-up assessments. Logistic regression models were used to analyze associations between lifestyle activity status and reversion from MCI to NC. We used a multiple adjustment model that was adjusted for demographic variables, primary diseases, lifestyle, and psychological variables as possible confounding factors in the participants with complete and imputed data sets. Adjusted odds ratios (ORs) for reversion and their 95% confidence intervals (CIs) were estimated. All analyses were performed using IBM SPSS v.25.0 (IBM Japan, Tokyo). Statistical significance was set at *P* < .05.

## Results

### Baseline characteristics of the participants

In the initial group of 396 participants, 202 participants (51.0%) reverted to NC from MCI. The reversion rate for participants for whom we imputed samples was 34.3%. Table 1 presents the measurements for participants with and without imputed samples. The two groups had the same mean scores and proportions except for one item regarding cognitive activity. Table 2 shows the participant baseline characteristics for those grouped according to the presence or absence of reversion from MCI to NC in the imputed group. Age, sex, educational level, smoking status, heart disease, pulmonary disease, hypertension, diabetes, walking speed, MMSE, GDS score, MCI status, and lifestyle activities differed significantly between participants with and without reversion (Table 2).

**Table 1:** Baseline characteristics in participants with complete data sets and participants for whom we imputed data

	Participants with complete data (*n* = 743)	Participants with imputed data (*n* = 37,893)
Age (years)*	71.8 (5.2)	71.8 (5.2)
Sex (% male)	53.8	53.8
Education (years)*	11.3 (2.5)	11.3 (2.5)
Current smoking (% yes)	10.6	10.6
Heart disease (% yes)	17.5	17.5
Pulmonary disease (% yes)	8.7	8.7
Hypertension (% yes)	47.5	47.5
Diabetes mellitus (% yes)	15.1	15.1
Walking speed (m/s)*	1.2 (0.2)	1.2 (0.2)
Mini-mental state examination (points)*	26.5 (1.8)	26.5 (1.8)
Geriatric depression scale (points)*	3.1 (2.6)	3.1 (2.6)
Category of MCI (% yes)		
Amnestic MCI single domain	14.3	14.3
Non-amnestic MCI single domain	62.2	62.2
Amnestic MCI multiple domain	6.7	6.7
Non-amnestic MCI multiple domain	16.8	16.8
Instrumental activities of daily living (% yes)		
Going outdoors using bus and train	89.3	89.3
Cash handling and banking	88.6	88.6
Driving a car	67.5	67.5
Using map to go unfamiliar place	57.9	57.9
Cognitive activity (% yes)		
Reading of book or newspaper	94.1	94.1
Cognitive stimulation such as board game and learning	44.2	44.2
Culture lesson	39.4	39.4
Using personal computer	28	26.8
Social activity (% yes)		
Daily conversation	95.4	95.4
Giving someone a helping hand	89.7	89.7
Attending a meeting in the community	52.4	52.4
Hobby or sports activity	69.4	69.4
Productive activity (% yes)		
Housecleaning	87.7	87.7
Field work or gardening	72.9	72.9
Taking care of grandchild or pet	54.4	54.4
Working	30.4	30.4

*Mean (standard deviation)

**Table 2:** Comparison of baseline characteristics between reverters and non-reverters

	Reverters (*n* = 12,897)	Non-reverters (*n* = 24,649)	*P* value
Age (years)	69.6 (3.6)	73.0 (5.6)	< 0.01
Sex (% male)	55.6	52.9	< 0.01
Education (years)	11.7 (2.4)	11.0 (2.5)	< 0.01
Current smoking (% yes)	9	11.4	< 0.01
Heart disease (% yes)	18.3	17.1	< 0.01
Pulmonary disease (% yes)	8.1	9.1	< 0.01
Hypertension (% yes)	43.4	49.6	< 0.01
Diabetes mellitus (% yes)	13.8	15.8	< 0.01
Walking speed (m/s)	1.3 (0.2)	1.2 (0.2)	< 0.01
Mini-mental state examination (points)	26.9 (1.8)	26.3 (1.8)	< 0.01
Geriatric depression scale (points)	2.7 (2.4)	3.3 (2.7)	< 0.01
Category of MCI			< 0.01
Amnestic MCI single domain	16.3	13.2	< 0.01
Non-amnestic MCI single domain	71.2	57.5	< 0.01
Amnestic MCI multiple domain	5.3	7.5	< 0.01
Non-amnestic MCI multiple domain	7.2	21.8	< 0.01
Instrumental activities of daily living (% yes)			
Going outdoors using bus and train	90.4	88.8	< 0.01
Cash handling and banking	89.1	88.4	0.08
Driving a car	76.8	62.7	< 0.01
Using map to go unfamiliar place	63.7	54.9	< 0.01
Cognitive activity (% yes)			
Reading of book or newspaper	96.5	92.9	< 0.01
Cognitive stimulation such as board game and learning	46.6	42.9	< 0.01
Culture lesson	43.3	37.4	< 0.01
Using personal computer	33.3	23.4	< 0.01
Social activity (% yes)			
Daily conversation	96.4	94.9	< 0.01
Giving someone a helping hand	91.2	89	< 0.01
Attending a meeting in the community	55.9	50.6	< 0.01
Hobby or sports activity	74.5	66.8	< 0.01
Productive activity (% yes)			
Housecleaning	88.5	87.3	< 0.01
Field work or gardening	74.6	72	< 0.01
Taking care of grandchild or pet	59	52	< 0.01
Working	33.8	28.7	< 0.01

### Associations between reversion and lifestyle activity

Table 3 shows the rate of reversion in participants who engaged in specific lifestyle activities. We found no significant relationships between reversion and lifestyle activities in the participants with complete data sets. In the participants for whom we imputed data, a logistic regression model showed that the odds ratios of reversion were significantly higher in participants who drove a car (OR 1.50, 95% CI 1.41–1.60, *P* < .01), used maps to travel to unfamiliar places (OR 1.12, 95% CI 1.06–1.18, *P* < .01), read books or newspapers (OR 1.54, 95% CI 1.37–1.73, *P* ≤ .01), participated in cultural classes (OR 1.10, 95% CI 1.04–1.15, *P* ≤ .01), attended meetings in the community (OR 1.22, 95% CI 1.16–1.28, *P* ≤ .01), participated in hobbies or sports activities (OR 1.09, 95% CI 1.03–1.16, *P* ≤ .01), and engaged in fieldwork or gardening (OR 1.14, 95% CI 1.08–1.21, *P* ≤ .01). The logistic models showed negative relationships between reversion and lifestyle activities that included cognitive stimulation, such as board games and learning (OR 0.92, 95% CI 0.88–0.97, *P* ≤ .01), daily conversations (OR 0.73, 95% CI 0.64–0.82, *P* ≤ .01), giving someone a helping hand (OR 0.76, 95% CI 0.70–0.83, *P* ≤ .01), and housecleaning (OR 0.90, 95% CI 0.84–0.98, *P* = .01).

**Table 3:** Odds ratios for mild cognitive impairment reversion according to lifestyle activity status (yes/no)

	Participants with complete data		Participants with imputed data	
	Odds ratio (95% CI)	*P* value	Odds ratio (95% CI)	*P* value
Instrumental activities of daily living (% yes)				
Going outdoors using bus and train	0.59 (0.25–1.41)	0.24	0.95 (0.87–1.02)	0.17
Cash handling and banking	1.04 (0.49–2.23)	0.92	0.97 (0.89–1.05)	0.39
Driving a car	1.65 (0.9–3.05)	0.11	1.50 (1.41–1.60)	< 0.01
Using map to go unfamiliar place	0.89 (0.52–1.52)	0.68	1.12 (1.06–1.18)	< 0.01
Cognitive activity (% yes)				
Reading of book or newspaper	1.73 (0.43–6.98)	0.44	1.54 (1.37–1.73)	< 0.01
Cognitive stimulation such as board game and learning	0.97 (0.59–1.6)	0.91	0.92 (0.88–0.97)	< 0.01
Culture lesson	0.83 (0.5–1.38)	0.47	1.10 (1.04–1.15)	< 0.01
Using personal computer	1 (0.57–1.75)	0.99	1.03 (0.97–1.09)	0.31
Social activity (% yes)				
Daily conversation	0.54 (0.16–1.9)	0.34	0.73 (0.64–0.82)	< 0.01
Giving someone a helping hand	0.53 (0.22–1.29)	0.16	0.76 (0.70–0.83)	< 0.01
Attending a meeting in the community	1.05 (0.64–1.73)	0.84	1.22 (1.16–1.28)	< 0.01
Hobby or sports activity	0.84 (0.47–1.47)	0.53	1.09 (1.03–1.16)	< 0.01
Productive activity (% yes)				
Housecleaning	0.98 (0.46–2.09)	0.96	0.90 (0.84–0.98)	0.01
Field work or gardening	1.07 (0.61–1.86)	0.82	1.14 (1.08–1.21)	< 0.01
Taking care of grandchild or pet	1.11 (0.69–1.79)	0.66	1.01 (0.96–1.06)	0.64
Working	0.91 (0.55–1.49)	0.70	1.02 (0.97–1.07)	0.55

### Associations between reversion and longitudinal change

Table 4 shows changes in activity patterns from the baseline to follow-up assessments. The proportion of non-reverters who started reading books or newspapers and stopped fieldwork or gardening was significantly higher than that of reverters during the 4-year follow-up period among the participants with complete data. The imputed sample showed that the proportion of non-reverters who began to drive a car, read books or newspapers, attended a meeting, or engaged in hobbies or sports increased significantly compared with reverters during the 4-year follow-up. However, non-reverters were significantly more likely to discontinue driving a car, read books or newspapers, and engage in hobbies or sports compared with reverters. The proportion of reverters who discontinued using maps, participation in cultural lessons, and attendance to meetings increased significantly compared with non-reverters in the imputed sample (Table 4).

**Table 4:** Changes in lifestyle activities between baseline and follow-up assessments

	Participants with complete data			Participants with imputed data		
	Reverters	Non-reverters	*P* value	Reverters	Non-reverters	*P* value
Driving a car			0.50			< .01
Unchanged	97.3	95.3	> .05	97.3	95.6	< .01
Begun	1.6	3.6	> .05	1.6	2.7	< .01
Discontinued	1.1	1.2	> .05	1.1	1.7	< .01
Using map to go unfamiliar place			.99			< .01
Unchanged	75.4	76.2	> .05	76.4	77.8	< .01
Begun	8	7.7	> .05	8.1	8.1	> .05
Discontinued	16.6	16.1	> .05	15.5	14.1	< .01
Reading of book or newspaper			.01			< .01
Unchanged	98.9	92.9	< .01	98.4	93.0	< .01
Begun	0	3.0	< .05	0.3	2.9	< .01
Discontinued	1.1	4.1	> .05	1.3	4.1	< .01
Culture lesson			.82			< .01
Unchanged	83.3	83.8	> .05	84.5	87.3	< .01
Begun	4.3	5.4	> .05	3.9	3.7	> .05
Discontinued	12.4	10.8	> .05	11.7	9.0	< .01
Attending a meeting in the community			.63			< .01
Unchanged	74.2	78.4	> .05	74.9	76.7	< .01
Begun	10.2	9.0	> .05	10.4	11.4	< .01
Discontinued	15.6	12.6	> .05	14.7	11.8	< .01
Hobby or sports activity			.54			< .01
Unchanged	81.3	76.5	> .05	81.5	77.8	< .01
Begun	7.5	14.5	> .05	7.2	8.7	< .01
Discontinued	11.2	9.0	> .05	11.3	13.5	< .01
Field work or gardening			.03			< .01
Unchanged	86.6	77.7	< .05	85.9	81.5	< .01
Begun	8.1	9.0	> .05	8.0	7.5	> .05
Discontinued	5.4	13.3	< .01	6.1	11.0	< .01

## Discussion

Although previous studies have reported many factors related to MCI reversion [9–13], information regarding reversible factors related to MCI reversion is limited. In this prospective study, reversion from MCI to NC was significantly associated with specific lifestyle activities after adjusting logistic regression models for many potential confounders. Participants were more likely to revert if they started driving a car, used maps to travel to unfamiliar places, read books or newspapers, participated in cultural lessons, attended community meetings, carried out hobbies or sports, and engaged in fieldwork or gardening among the participants with imputed data. Driving cessation is associated with a number of negative consequences, such as declined general health [35], cognitive decline [36], depressive symptoms [37], increased risk for long-term care institutionalization [38], and mortality [39]. The results suggest that driving is associated with maintenance of cognitive function. Additionally, increasing the amount of time spent driving may promote the use of maps, which is likely beneficial for improving cognitive function. In terms of cognitive activity, our work is consistent with previous findings indicating that participation in cognitive activities is associated with reduced rates of cognitive decline in elderly people [40, 41]. However, we found that specific cognitive activities, such as reading books or newspapers, were particularly associated with reversion from MCI to NC. Low levels of social and IADL activities are considered to predict future functional decline among community-dwelling older people [42], and human interaction has a positive influence on mental health [43]. Meetings and hobbies or sports are social activities that include human interaction. Thus, it is unsurprising that these activities are related to MCI reversion. In terms of productive activities, countrywide and population-based research has indicated that gardening may be beneficial for adult mental health, including self-rated health and psychological distress [44]. Our findings regarding the relationship between gardening or fieldwork and MCI reversion in older populations support these previous results. Although some lifestyle activities were associated positively with reversion from MCI to NC, board games and learning, daily conversation, giving someone a helping hand, and housecleaning were negatively correlated with MCI reversion. The implementation rates of these activities (with the exception of board games) were high, about 90%, which may have led to inconsistent results with respect to previous studies.

Regarding changes in lifestyle activities between baseline and follow-up assessments, more non-reverters discontinued driving (1.7% vs. 1.1%), reading books or newspapers (4.1% vs. 1.3%), and participation in hobbies or sports activities (13.5% vs. 11.3%) during the study period, while more non-reverters increased engagement in these activities. Thus, the evidence is not sufficiently persuasive to recommend initiation of these activities or an increase in participation for the purpose of preventing decline or reversing MCI. However, more non-reverters discontinued fieldwork or gardening (11.0% vs. 6.1%) during the study period and non-reverters did not increase engagement in these activities. Thus, it is reasonable to recommend continuation of fieldwork or gardening for recovery from MCI.

This study had several limitations. Previous research using cluster analytic techniques has demonstrated that MCI participants can be grouped based on similarities in their neuropsychological profiles, providing a method of describing MCI subtypes without being confined to the amnestic/non-amnestic distinction [45, 46]. One critical finding from a cluster analytic study was the identification of a large subgroup who performed within normal limits on cognitive testing despite their MCI diagnosis [45]. The cluster-derived normal group comprised one third (34%) of the Alzheimer’s Disease Neuroimaging Initiative MCI sample and was comparable to a robust normal control group in cognitive test performance and percentage of individuals with positive cerebrospinal fluid biomarkers for AD [47]. In addition, the cluster-derived normal group had fewer APOE-ε4 carriers and fewer individuals with positive CSF biomarkers of AD than the MCI groups and was also less likely to progress to AD and more likely to revert to normal than the MCI groups [47]. These results suggest that the conventional diagnosis of MCI may be highly susceptible to false positive diagnostic errors, which is consistent with previous reports of high reversion rates or lack of progression in those with MCI [3]. The high reversion rate in the study may be affected by the false positive issue, and it is difficult to accurately determine the influence of activities on reversion MCI to NC. And the participants were not randomly recruited, and about 47% of the participants dropped out at the follow-up. This dropout rate may have led to underrepresentation of cognitive decline and dementia. Moreover, we did not collect data about potential correlates, such as nutritional status, which could affect MCI reversion.

One strength of the present study is that our findings are consistent with comprehensive geriatric assessments designed to collect information about lifestyle activity. In addition, to our knowledge, this is the first study to use a large population-based sample to examine correlations between specific lifestyle activities and MCI reversion.

## Conclusions

The present results indicate that reversion from MCI to NC was significantly associated with specific lifestyle activities including driving a car, using maps to travel to unfamiliar places, reading books or newspapers, taking culture lessons, attending community meetings, participating in hobbies or sports activities, and doing fieldwork or gardening among the participants with imputed samples. The longitudinal data indicate that continuing fieldwork or gardening may increase the chance of recovery from MCI.

## Additional file

Additional file 1: Baseline characteristics in participants who remained versus left the follow-up. (DOCX 18 kb)

## Abbreviations

AD: Alzheimer’s disease
CI: Confidence interval
GCI: global cognitive impairment
GDS: Geriatric Depression Scale
IADL: Instrumental activities of daily living
MCI: Mild cognitive impairment
MMSE: Mini-Mental State Examination
NC: Normal cognition
NCGG-FAT: National Center for Geriatrics and Gerontology-Functional Assessment Tool
NCGG-SGS: National Center for Geriatrics and Gerontology – Study of Geriatric Syndromes
OR: Odds ratio
OSHPE: Obu Study of Health Promotion for the Elderly
SD: Standard deviation
TMT: Trail Making Test
