# Supplementary material for: Reversible predictors of reversion from mild cognitive impairment to normal cognition: a 4-year longitudinal study
Source: Alzheimers Res Ther. 2019 Mar 13;11:24. doi: 10.1186/s13195-019-0480-5 (PMC6416893; doi:10.1186/s13195-019-0480-5)
Supplement: Supplementary file 1 — Baseline characteristics in participants who remained versus left the follow-up. (DOCX 18 kb) [file 13195_2019_480_MOESM1_ESM.docx]

# Additional file 1. Baseline characteristics in participants who remained versus left the follow up.

|  | Participants who remained the follow-up (n = 396) | Participants who left the follow-up (n =347) | P value |
| --- | --- | --- | --- |
| Age (years) | 71.1 (4.5) | 72.7 (5.8) | <.01 |
| Sex (% male) | 45.7 | 46.7 | >.05 |
| Education (years) | 11.5 (2.4) | 10.9 (2.5) | <.01 |
| Current smoking (% yes) | 9.3 | 12.1 | >.05 |
| Heart disease (% yes) | 16.4 | 18.7 | >.05 |
| Pulmonary disease (% yes) | 8.8 | 8.6 | >.05 |
| Hypertension (% yes) | 45.2 | 50.1 | >.05 |
| Diabetes mellitus (% yes) | 15.2 | 15.0 | >.05 |
| Walking speed (m/s) | 1.3 (0.2) | 1.2 (0.2) | <.01 |
| Mini-mental state examination (points) | 26.6 (1.9) | 26.5 (1.8) | >.05 |
| Geriatric depression scale (points) | 2.9 (2.6) | 3.2 (2.7) | >.05 |
| **Category of MCI** |  |  | >.05 |
| amnestic MCI single domain | 13.6 | 15.0 |  |
| non-amnestic MCI single domain | 62.1 | 62.2 |  |
| amnestic MCI multiple domain | 7.8 | 5.5 |  |
| non-amnestic MCI multiple domain | 16.4 | 17.3 |  |
| **Instrumental activities of daily living (% yes)** |  |  |  |
| Going outdoors using bus and train | 91.4 | 87.0 | <.05 |
| Cash handling and banking | 89.6 | 87.6 | >.05 |
| Driving a car | 73.0 | 61.3 | <.01 |
| Using map to go unfamiliar place | 63.9 | 51.0 | <.01 |
| **Cognitive activity (% yes)** |  |  |  |
| Reading of book or newspaper | 96.7 | 91.1 | <.01 |
| Cognitive stimulation such as board game and learning | 45.2 | 43.1 | >.05 |
| Culture lesson | 44.8 | 33.1 | <.01 |
| Using personal computer | 29.8 | 23.3 | <.05 |
| **Social activity (% yes)** |  |  |  |
| Daily conversation | 96.2 | 94.5 | >.05 |
| Giving someone a helping hand | 91.4 | 87.8 | >.05 |
| Attending a meeting in the community | 58.4 | 45.5 | <.01 |
| Hobby or sports activity | 73.5 | 64.6 | <.01 |
| **Productive activity (% yes)** |  |  |  |
| Housecleaning | 87.9 | 87.6 | >.05 |
| Field work or gardening | 76.2 | 69.1 | <.05 |
| Taking care of grandchild or pet | 56.5 | 52.0 | >.05 |
| Working | 33.1 | 27.4 | >.05 |

* Mean (Standard Deviation)
